# Recurrent atypical teratoid/rhabdoid tumors (AT/RT) reveal discrete features of progression on histology, epigenetics, copy number profiling, and transcriptomics

**DOI:** 10.1007/s00401-023-02608-7

**Published:** 2023-07-14

**Authors:** Pascal D. Johann, Lea Altendorf, Emma-Maria Efremova, Till Holsten, Mona Steinbügl, Karolina Nemes, Alicia Eckhardt, Catena Kresbach, Michael Bockmayr, Arend Koch, Christine Haberler, Manila Antonelli, John DeSisto, Martin U. Schuhmann, Peter Hauser, Reiner Siebert, Susanne Bens, Marcel Kool, Adam L. Green, Martin Hasselblatt, Michael C. Frühwald, Ulrich Schüller

**Affiliations:** 1 Paediatric and Adolescent Medicine, Swabian Children’s Cancer Center Augsburg, EU-RHAB Trial Center, Germany and Bavarian Cancer Research Center (BZKF), Augsburg, Germany; 2 Hopp Children’s Cancer Center (KiTZ), Heidelberg, Germany; 3 Division of Pediatric Neurooncology, German Cancer Research Center (DKFZ) and German Cancer Research Consortium (DKTK), Heidelberg, Germany; 4 Department of Pediatric Hematology and Oncology, University Medical Center Hamburg-Eppendorf, Hamburg, Germany; 5 Research Institute Children’s Cancer Center Hamburg, Martinistraße 52, N63, 20251 Hamburg, Germany; 6 Institute of Neuropathology, University Medical Center Hamburg-Eppendorf, Hamburg, Germany; 7 Department of Radiotherapy and Radio-Oncology, University Medical Center Hamburg-Eppendorf, Hamburg, Germany; 8 Mildred Scheel Cancer Career Center HaTriCS4, University Medical Center Hamburg-Eppendorf, Hamburg, Germany; 9 Institute of Neuropathology, Charité, Universitätsmedizin Berlin, Berlin, Germany; 10 Institute of Neurology, Medical University of Vienna, Vienna, Austria; 11 Comprehensive Cancer Center, Medical University of Vienna, Vienna, Austria; 12 Department of Radiological, Oncological and Anatomic Pathology Sciences, Università Sapienza, Rome, Italy; 13 Morgan Adams Foundation Pediatric Brain Tumor Research Program, Children’s Hospital Colorado, Aurora, CO USA; 14 Department of Pediatrics, University of Colorado Denver, Aurora, CO USA; 15 Division of Pediatric Neurosurgery, Department of Neurosurgery, Eberhard Karl’s University Hospital of Tübingen, Tübingen, Germany; 16 Second Department of Pediatrics, Semmelweis University, Budapest, Hungary; 17 Institute of Human Genetics, Ulm University & Ulm University Medical Center, Ulm, Germany; 18 Princess Máxima Center for Pediatric Oncology, Utrecht, The Netherlands; 19 Institute of Neuropathology, University Hospital Münster, Münster, Germany

**Keywords:** AT/RT, Rhabdoid tumor, Pediatric cancer, Recurrent tumor, DNA methylation, RNA sequencing

## Abstract

Atypical teratoid/rhabdoid tumors (AT/RT) are the most common malignant brain tumors manifesting in infancy. They split into four molecular types. The major three (AT/RT-SHH, AT/RT-TYR, and AT/RT-MYC) all carry mutations in *SMARCB1*, the fourth quantitatively smaller type is characterized by *SMARCA4* mutations (AT/RT-SMARCA4). Molecular characteristics of disease recurrence or metastatic spread, which go along with a particularly dismal outcome, are currently unclear. Here, we investigated tumor tissue from 26 patients affected by AT/RT to identify signatures of recurrences in comparison with matched primary tumor samples. Microscopically, AT/RT recurrences demonstrated a loss of architecture and significantly enhanced mitotic activity as compared to their related primary tumors. Based on DNA methylation profiling, primary tumor and related recurrence were grossly similar, but three out of 26 tumors belonged to a different molecular type or subtype after second surgery compared to related primary lesions. Copy number variations (CNVs) differed in six cases, showing novel gains on chromosome 1q or losses of chromosome 10 in recurrences as the most frequent alterations. To consolidate these observations, our cohort was combined with a data set of unmatched primary and recurrent AT/RT, which demonstrated chromosome 1q gain and 10 loss in 18% (*n* = 7) and 11% (*n* = 4) of the recurrences (*n* = 38) as compared to 7% (*n* = 3) and 0% (*n* = 0) in the primary tumors (*n* = 44), respectively. Similar to the observations made by DNA methylation profiling, RNA sequencing of our cohort revealed AT/RT primary tumors and matched recurrences clustering closely together. However, a number of genes showed significantly altered expression in AT/RT-SHH recurrences. Many of them are known tumor driving growth factors, involved in embryonal development and tumorigenesis, or are cell-cycle-associated. Overall, our work identifies subtle molecular changes that occur in the course of the disease and that may help define novel therapeutic targets for AT/RT recurrences.

**Supplementary Information:**

The online version contains supplementary material available at 10.1007/s00401-023-02608-7.

## Introduction

Malignant rhabdoid tumors (MRTs) mainly affect infants and young children. They are known for their highly aggressive behaviour and dismal prognosis with 5-year overall survival (OS) ranging from 0% to 78% depending on risk groups [11, 30]. Most commonly, they are located in the central nervous system (CNS, 66%), where they are termed atypical teratoid/rhabdoid tumors (AT/RT).

AT/RT may be divided into four molecular types, which differ in their clinical behaviour, genetics, epigenetic signature, and gene expression profile. The three main types are AT/RT-SHH, AT/RT-TYR, AT/RT-MYC and AT/RT-SMARCA4 is only rarely represented [16, 17, 29]. AT/RT-SHH tumors can further be divided into three subtypes that differ by localization, clinical outcome and methylation profile: AT/RT-SHH-1A, AT/RT-SHH-1B, and AT/RT-SHH-2 [9]. In 95% of all AT/RT, the underlying genetic cause is a mutation of the *SMARCB1* gene [3]. SMARCB1 is part of the SWItch/sucrose non-fermentable (SWI/SNF) complex, which is involved in gene regulation and tumor development [31]. *SMARCB1* mutations are already present in the germline in up to 30% of patients, who then suffer from a rhabdoid tumor predisposition syndrome 1 (RTPS1; OMIM #609322) [10, 25]. In rare cases, the *SMARCA4* gene is affected instead [14, 24] (AT/RT-SMARCA4). If the mutation is already presented in the germline, patients suffer from a rhabdoid tumor predisposition syndrome 2 (RTPS2; OMIM #613325) [10, 25]. Patients with tumors of this molecular type carry germline mutations even more often than patients with *SMARCB1* mutated AT/RT, present at younger age, and suffer from an inferior prognosis with shorter survival times. The best prognosis is observed for AT/RT-TYR, especially when patients are older than 12 months [11, 30].

The treatment of AT/RT is challenging, and progression is inevitabley observed, if the tumor is non-responsive to therapy. Even if patients reach remission following gross total resection, tumors recur in up to 50% of patients. In fact, only very few of patients suffering from recurrences are still alive 5 years following diagnosis of a recurrence [8, 26]. Molecular alterations, which are responsible for or contribute to tumor progression or recurrence, are currently largely unknown. It will be helpful to identify these molecular alterations and thus possible therapeutic targets for patients with recurrent tumors. Biopsies of AT/RT recurrences are only rarely available. This has impeded the research on specific histological and molecular features and the development of targeted therapies in the past.

In this study, samples of a relatively large and unique cohort of 26 patients, all suffering from recurrent AT/RT, were analyzed. We summarize the clinical course of the patients and provide in-depth molecular data comparing primary and recurrent tumor samples using histological analysis, DNA methylation profiling, and RNA sequencing.

## Materials and methods

### Data sets

Clinical information was derived from the databases of the EU-RHAB registry. EU-RHAB has received continuous ethical approval (Ethics Committee University of Münster, Germany 2009-532-f-S, most recently amended 17AUG2021). Genomic, histological, DNA methylation, and RNA sequencing data were collected from the different laboratories Hamburg, Heidelberg, Denver, Kiel, and Ulm. Resampling was performed during autopsy for cases 13, 19, 20, and 21, and via biopsy or resection in all other cases (Table 1). Reference data sets consist of unpublished or published data summarized in Supplementary Tables 1 and 2.

**Table 1:** Molecular and clinical data of all analyzed patients with samples for primary AT/RT and recurrences (*n* = 26)

Case No	Sex	Age of onset [years]	Localization	Molecular type and subtype	Resection status	Pathogenic* SMARCB1* germline variant	Time to first re-sampling [months]	Therapy protocol	Follow-up [months]	Status
1	m	5.0	Supratentorial	SHH-1A	Subtotal resection	Wild type	30.5	Rhabdoid 07	70.5	Dead
2	m	1.8	Supratentorial	SHH-1B	Subtotal resection	Wild type	41.0	EU-RHAB	57.4	Alive
3	m	1.6	Supratentorial	TYR	Subtotal resection	wild Type	25.5	Rhabdoid 07	59.2	Dead
4	m	0.1	Infratentorial	TYR	Subtotal resection	Wild type	5.0	EU-RHAB	12.0	Dead
5	f	2.0	Supratentorial	SHH-1B	n.a.	n.a.	3.3	HIT SKK 87	10.6	Dead
6	m	2.0	Supratentorial	MYC	Subtotal resection	n.a.	1.5	EU-RHAB	6.1	Dead
7	f	0.4	Infratentorial	SHH-2	Subtotal resection	c.170delTG	4.5	Rhabdoid 07	23.3	Dead
8	m	0.1	Infratentorial	TYR	Subtotal resection	Wild type	4.0	EU-RHAB	10.0	Dead
9	f	1.0	Infratentorial	TYR	Gross total resection	Wild type	5.3	Rhabdoid 07	85.3	Alive
10	m	2.0	Infratentorial/spinal	MYC/SHH-2	Subtotal resection	c.118C > T	n.a.	EU-RHAB	90.0	Alive
11	m	5.0	Supratentorial	SHH-1A	n.a.	Wild type	16.5	n.a.	99.2	Alive
12	f	0.9	Infratentorial	SHH-2/SHH-1A	Subtotal resection	c.157C > T p.R53X	48.0	EU-RHAB	48.0	Dead
13	m	10.0	n.a.	MYC	n.a.	c.157C > T p.Arg53*	n.a.	n.a.	n.a.	Dead
14	m	2.1	Infratentorial	TYR	Subtotal resection	Wild type	5.5	EU-RHAB	24.0	Dead
15	m	2.2	Supratentorial	SHH-1B	Gross total resection	n.a.	6.0	EU-RHAB	45.0	Dead
16	m	5.0	Supratentorial	SHH-1A	Subtotal resection	Wild type	32.5	EU-RHAB	9.0	Dead
17	f	1.0	Supratentorial	TYR	n.a.	n.a.	10.0	n.a.	n.a.	n.a.
18	m	2.5	Supratentorial	MYC	Gross total resection	Yes	11.0	Individual	17.6	Dead
19	m	2.7	Supratentorial	SHH-1B	Subtotal resection	n.a.	18.0	DFCI protocol	18.0	Dead
20	m	3.2	Supratentorial	SHH-1A	Subtotal resection	n.a.	15.0	DFCI protocol	15.0	Dead
21	f	1.3	Supratentorial	SHH-2/SHH-1B	Biopsy	c.118C > T	10.0	Individual	10.0	Dead
22	m	0.7	Infratentorial	MYC	Gross total resection	n.a.	6.0	Individual	6.0	Dead
23	m	0.6	Supratentorial	SHH-1A	n.a.	Wild type	6.7	EU-RHAB	16.0	Dead
24	m	1.4	Infratentorial	SMARCA4	n.a.	Wild type^a^	n.a.	n.a.	28.0	Dead
25	f	2.0	Supratentorial	SHH-1A	Subtotal resection	yes	37.0	EU-RHAB	37.9	Alive
26	m	1.2	Infratentorial	TYR	n.a.	yes	5.0	n.a.	5.0	Alive

Table including characteristics of all cases with paired primary tumors and recurrences. The cases 1, 6, and 7 had two recurrences and the case 7 also two primary tumors. Molecular type of each tumor was determined using brain tumor classifier (v12.5) (www.molecularneuropathology.org) and AT/RT-SHH subtypes were determined according to *t*-SNE plot (Fig. 3b). AT/RT-SMARCA4 was identified by *SMARCA4* mutation. Types or subtypes of cases 10, 12, and 21, and localization of case 10 are listed as primary tumor/recurrence

*m* male, *f* female, *n.a.* not available, *DOX* doxorubicin, *ICE* ifosfamide, carboplatinum, etoposide, *VCA* vincristine, cyclophosphamide, actinomycin, *VCD* vincristine, cyclophosphamide, doxorubicin, *TMZ* temozolomide, *DTIC* dacarbazine

^a^Pathogenic *SMARCA4* germline variant (c.2335 G > A)

Re-sampling was performed during autopsy for cases 13, 19, 20, 21 and via biopsy or resection in all other cases. Therapy according to EU-RHAB included either 9 alternate courses (3 × DOX, ICE, VCA) or 6 alternate courses (2 × DOX, ICE, VCA), followed by high-dose chemotherapy with Carboplatin/Thiotepa, with both arms involving intraventricular methotrexate (MTX) until start of radiotherapy (RTX). RTX was administered if the patient was older than 18 months of age. Rhabdoid 2007 included 9 cycles of chemotherapy (5 × VCD and 4 × ICE) and intraventricular chemotherapy (MTX or MTX/cytarabine/hydrocortisone) until start of RTX. RTX was only applied to children of more than 18 months of age. The HIT SKK 87 trial involved procarbazine, ifosfamide, etoposide, MTX, cytarabine and cisplatinum as induction therapy given in two cycles, followed by maintenance therapy with procarbazine, MTX and vincristine until start of RTX. The Dana Farber Cancer Institute (DFCI) protocol consisted of a modified IRS-III regimen involving VCR, ACT D, cyclophosphamide, cisplatin, DOX, and TMZ in lieu of DTIC as well as intrathecal chemotherapy with MTX, cytarabine and hydrocortisone and focal (M0) or CSI (M +) RTX

### Histology and immunohistochemistry

Tumor samples were fixed in 4% formaldehyde for 24 h. Subsequently, they were dehydrated, embedded in paraffin, and sectioned at 4 µm. Hematoxylin and Eosin (H&E) staining was performed using standard protocols. Immunohistochemical stainings were done using a Ventana System (Roche) according to manufacturers’ specifications. The antibodies rabbit anti-Ki67 (Abcam #ab16667, 1:200) and mouse anti-BAF47 (BD Biosciences #612110, 1:50) were used for Ki67 and SMARCB1 stainings. For 19 patients, the mitotic figures of ten high power fields (HPF) were counted for each primary tumor and related recurrence with subsequent comparison of the averages. Ki67-positive nuclei of 16 cases were counted and the frequency was compared between primary and recurrent AT/RT. Significance was determined using a *t* test for paired data. SMARCB1-deficiency was identified by immunohistochemical staining using anti-SNF5/SMARCB1 antibody (Abcam #ab88589, 1:50). For SMARCA4-deficient cases, anti-BRG1 antibody (Abcam #ab110641, 1:25) was used.

### DNA methylation profiling

The DNA of the tumor samples from FFPE tissue was isolated using “Maxwell® FFPE DNA Kit” (Promega) according to manufacturer’s specifications. For KRYO-conserved samples, the DNA was isolated using the “High Pure PCR Template Preparation Kit” (Roche). Bisulfite conversion was performed with the “EZ DNA Methylation Kit” (Zymo Research) using 100–500 ng DNA. FFPE DNA was cleaned and converted using the “DNA Clean & Concentrator-5 Kit” (Zymo Research) and the “Infinium HD FFPE DNA Restore Kit” (Illumina). This cleaning and conversion step was not necessary for KRYO DNA. In a final step, the methylation status of 450,000 or 850,000 CpG sites was analyzed on an “iScan device” (Illumina) after hybridizing the DNA to a “HumanMethylation450” or a “MethylationEPIC” BeadChip array (Illumina), respectively.

### Identification of molecular types based on DNA methylation data

The tumor entity and molecular types AT/RT-SHH, AT/RT-MYC, and AT/RT-TYR were identified using the brain tumor classifier (v12.5, www.molecularneuropathology.org), which is a platform for DNA methylation-based classification of CNS tumors. Results were validated by *t*-stochastic neighbour embedding (*t*-SNE) analysis. So far, the classifier cannot identify AT/RT-SMARCA4 or AT/RT-SHH subtypes. AT/RT-SHH-1A, AT/RT-SHH-1B and AT/RT-SHH-2 were allocated to our cases based on a *t*-SNE analysis. AT/RT-SMARCA4 cases were identified by *SMARCA4* mutation. Therefore, classifier scores only refer to AT/RT-SHH, AT/RT-MYC, and AT/RT-TYR. To demonstrate how the 26 cases of our cohort fit into the landscape of established AT/RT subtypes, we also included a large cohort of reference cases from all known molecular types (*n* = 138). Reference data sets consist of unpublished or published data summarized in Supplementary Tables 1 and 2.

### RNA sequencing

RNA isolation from FFPE and KRYO tissue samples was performed using “Maxwell® RSC RNA FFPE Kit” (Promega). An “RNA 6000 Nano Chip” was inserted on an “Agilent 2100 Bioanalyzer” (Agilent Technologies) to analyze RNA integrity. Next, depletion of ribosomal RNA was performed using the “RiboCop rRNA Depletion Kit” (Lexogen). Subsequently, the “CORALL Total RNA-Seq Library Prep Kit” (Lexogen) was used for the generation of a RNA sequencing library. Concentration measurement was performed for all samples with the “Qubit 2.0 Fluorometer” (Thermo Fisher Scientific) and the distribution of the final libraries’ fragment lengths was determined using the “DNA High Sensitivity Chip”, again on an “Agilent 2100 Bioanalyzer” (Agilent Technologies). The concentration of all samples was adjusted to 2 nM to pool them at equimolar concentrations. Sequencing of the library pool was done on a “NextSeq500” (Illumina) with 1 × 75 bp, with 24.5 to 35.1 million reads per sample.

### Bioinformatics and statistics of DNA methylation data

DNA methylation analysis was performed using raw IDAT files resulting from DNA methylation profiling. The files were processed and visualized using “R Studio” (version 4.1.3). IDAT files were loaded using the *minfi* package (version 1.40.0). Data from 450 k and EPIC arrays were combined and preprocessed using the *Noob* function for normalization. All probes containing SNPs within two nucleotides around a CpG site were removed as well as probes targeting the sex chromosomes. Probes which did not explicitly target the human genome were removed from analysis. Batch effects introduced due to different array and conservation types were removed using *ComBat*-function from *sva* R package (version 3.42.0). To make sure that primary tumors and related recurrences belonged to the same patient, a genotype matching was performed. By identification of common SNPs, we could ensure if primary tumor and recurrence belonged to one patient. Finally, the 1000 CpG sites with the largest standard deviation across all patient samples were chosen for further analysis of DNA methylation data. A heatmap was created using the R package *ComplexHeatmap* (version 2.10.0). The clustering distance metric for columns and rows was defined as “Pearson”. In addition, the clustering method for columns and rows was set as “average”. A *t*-SNE analysis was implemented using the R package *Rtsne* (version 0.16)). The values for the *t*-SNE analysis were calculated by defining the “eigenvalue k” as “*k* = 50”, and 100 random numbers were created using “set.seed(100)”. The arguments for *Rtsne* function were set as “max_iter = 2000” and “perplexity = 20”. Changes of molecular type or subtype were visualized in an alluvial plot using *ggalluvial* package. Primary-recurrence variation was defined as the Jensen–Shannon divergence (JSD) between recurrences and related primary tumors based on the 1000 CpG sites with the largest standard deviation using the *philentropy* R package. Significant differences in global DNA methylation patterns for all CpG sites were determined using a Wilcoxon test for independent samples. For methylation-derived copy number variations (CNV), the individual copy number profiles were calculated based on raw methylated/unmethylated signals using the *conumee* R package (version 1.28.0). Ratios were normalized against reference samples and cutoff using the local (arm segment) threshold 0.25 and -0.25, respectively.

### Bioinformatics and statistics of RNA sequencing data

Raw RNA sequencing count data were used as input for the analysis in “R Studio” (version 4.1.3). As a first step, matching HUGO symbols for the ENSEMBL IDs were added using the *biomaRt* package (version 2.52.0). Repetitively counted genes were identified and removed as well as genes from sex chromosomes. Batch effects due to different conservation types and the integration of reference cases were corrected using *ComBat_Seq* function from *sva* package (version 3.44.0). In the next step, the *DESeq2* package (version 1.36.0) was used to perform *DESeq2* analysis of raw count data. The resulting data were normalized using variance stabilized transformation (VST). Finally, the 1000 most variable genes, identified by standard deviation, were chosen for cluster analysis. A heatmap and a *t*-SNE plot were created as described for the DNA methylation data. Primary-recurrence variation was defined as the Jensen–Shannon divergence (JSD) between recurrences and related primary tumors based on the 1000 most variable genes using the *philentropy* R package. For the selection of the differentially expressed genes, all genes, identified by *DESeq2* analysis, with a significant *p* value (*p*_adjusted_ < 0.05) after Benjamini–Hochberg (BH) adjustment and a log_2_ fold change (log_2_ FC) > 1 were chosen. This was done for each molecular type, separately. Differentially expressed genes between primary and recurrent AT/RT were visualized in volcano plots created with the *EnhancedVolcano* package (version 1.14.0). Log_2_ of *DESeq2*-normalized counts of three upregulated and three downregulated genes in recurrences was compared to matched primary tumors. Statistical analysis was performed using *DESeq2*. Pathway analyses were performed using the *gsePathway* function of the *ReactomePA* package (version 1.40.0) with a gene list ranked by log_2_ FC as input and Reactome pathways. The results were visualized using *cnetplot* function of the *enrichplot* package (version 1.16.2).

### Immune cell infiltration

The infiltration of immune cells in the present tumor samples was quantified based on raw DNA methylation data and RNA sequencing count data. The estimation of the infiltration with tumor-infiltrating lymphocytes (TILs), CD4^+^ T cells, and CD8^+^ T cells was performed by calculation of the DIMEimmune scores according to Safaei et al. [23] using DNA methylation data. The infiltration with additional cell populations was computed from RNA sequencing data using a deconvolution method optimized for brain tumors [4, 5]. This method quantified the infiltration with T cells, CD8^+^ T cells, cytotoxic lymphocytes, B lineage cells, NK cells, monocytic lineage cells, myeloid dendritic cells, neutrophils, endothelial cells, and fibroblasts. Significant differences were determined using a *t*-test for paired data.

### Data Availability

The DNA methylation and RNA sequencing data of paired primary and recurrent AT/RT of this study are deposited at NCBI Gene Expression Omnibus (GEO; https://www.ncbi.nlm.nih.gov/geo). They are accessible through GEO Series accession numbers GSE228091 (DNA methylation) and GSE228101 (RNA sequencing).

## Results

### Assembly of a cohort of 26 patients with primary AT/RT and tissue from matched recurrences

**Fig. 1:**
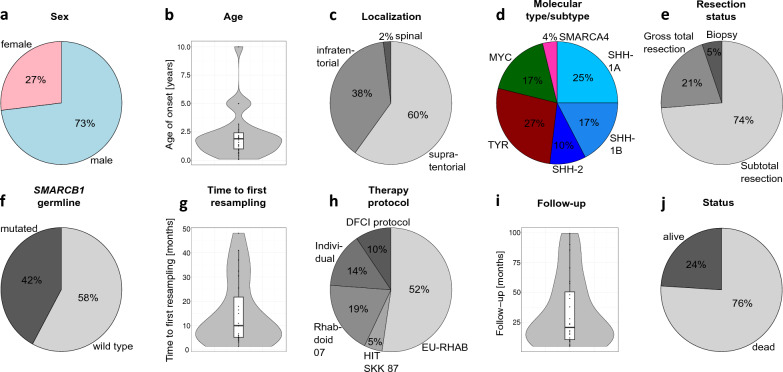
Overview of molecular and clinical data referring to primary tumors of all analyzed patients with samples for primary AT/RT and recurrences (*n* = 26). **a** Proportion of the gender (*n* = 26/26). **b** Age distribution in years at diagnosis (*n* = 26/26). **c** Proportion of the localization of primary and recurrent AT/RT (*n* = 50/52). **d** Proportion of the molecular types and subtypes in primary tumors and recurrences (*n* = 52/52). **e** Proportion of the resection status after surgery of the primary AT/RT (*n* = 19/26). **f** Proportion of pathogenic *SMARCB1* germline variants (*n* = 19/26). **g** Distribution of time of the first resampling in months (*n* = 23/26). **h** Proportion of therapy protocols (*n* = 21/26). **i** Distribution of the follow-up in months (*n* = 24/26). **j** Proportion of alive and dead patients (*n* = 25/26)

A cohort of 26 patients with primary AT/RT and matched recurrences are the basis of the current study (Table 1, Fig. 1). DNA methylation profiling classified them as AT/RT-SHH, AT/RT-TYR, and AT/RT-MYC using brain tumor classifier and the subtypes AT/RT-SHH-1A, AT/RT-SHH-1B, and AT/RT-SHH-2 were assigned using *t*-SNE analysis. AT/RT-SMARCA4 was identified by *SMARCA4* mutation. The patients #1, 6, and 7 had two recurrences, and case 7 included two primary tumor samples. Each primary tumor and related recurrence had the same mutations in *SMARCB1* or *SMARCA4* (Table 1) and genotype matching confirmed that they belonged to one patient based on the identification of common SNPs. Most of the AT/RT pairs (73%, *n* = 19/26) were from male patients (Fig. 1a), and average age of disease onset was 2.2 years (Fig. 1b). Patients, who were older than or equal to 5 years (*n* = 4/26) belonged exclusively to the AT/RT-SHH or AT/RT-MYC types, whereas patients with AT/RT-TYR tumors were very young with six out of seven cases being younger than 2 years at disease onset (Table 1). The majority of primary tumors and recurrences (60%, *n* = 30/50) was located supratentorially, 38% (*n* = 19/50) were infratentorial, and the recurrence of patient #10 was spinal (2%, *n* = 1/50, Fig. 1c). Five out of seven AT/RT-TYR and two out of three AT/RT-SHH-2 cases were localized infratentorially (Table 1). A proportion of 25% (n = 13/52) of all tumors belonged to the AT/RT-SHH-1A subtype, 17% (*n* = 9/52) to AT/RT-SHH-1B, 10% (*n* = 5/52) to SHH-2, 27% (*n* = 14/52) to AT/RT-TYR, 17% (n = 9/52) to AT/RT-MYC, and 4% (*n* = 2/52) to AT/RT-SMARCA4 (Fig. 1d). All of these observations and distributions are in line with the current literature [9, 11, 15, 16].

Furthermore, in most of the patients a subtotal resection of their primary AT/RT was achieved (74%, *n* = 14/19), whereas approximately a quarter received gross total resections (21%, *n* = 4/19). For one patient only a biopsy was performed (5%, *n* = 1/19, Fig. 1e). The single patient with the AT/RT-SMARCA4 tumor and almost half of all analyzed patients (42%, *n* = 8/19) revealed a pathogenic germline variant (Fig. 1f). The average time to first resampling (*n* = 23) was 15.1 months (Fig. 1g). Approximately half of the patients received a therapeutic approach according to EU-RHAB recommendations (52%, *n* = 11/21), whereas the other patients received therapies according to the Dana Farber Cancer Institute (DFCI) protocol (10%, *n* = 2/21), Rhabdoid 2007 (19%, n = 4/21), and HIT SKK 87 trial (5%, *n* = 1/21). An individual therapy was applied in the cases of three patients (14%, *n* = 3/21, Fig. 1h). The average follow-up of the patients was 33.5 months (Fig. 1i) with 76% (n = 19/25) of the patients having passed away at the time of data acquisition (Fig. 1j).

### Histology of recurrent AT/RT reveals increased mitotic activity and loss of architecture

**Fig. 2:**
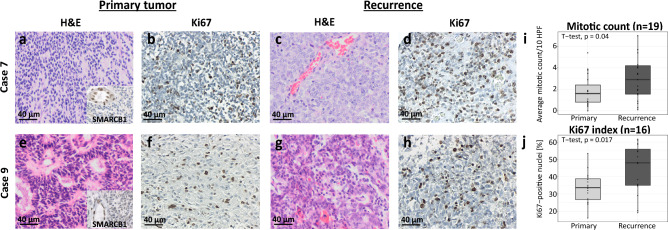
Histological features of recurrent AT/RT compared to primary tumors. Recurrent tumors demonstrate loss of architecture, increased number of mitotic figures using H&E staining (**a**, **c**, **e**, **g**), and intensified Ki67 labeling (**b**, **d**, **f**, **h**). All samples showed negative SMARCB1 staining (**a**, **e**: square box below right), confirming the diagnosis of AT/RT. **i** Mitotic activity increased in recurrent tumors. **j** Ki67 index was heightened in recurrent AT/RT compared to primary tumors. *HPF* high-power-field

The primary tumors and their recurrences (Table 1) were analyzed histologically using microscopy of H&E and Ki67 staining. As opposed to primary specimens (Fig. 2a, e), H&E-stained recurrences revealed a loss of architecture with features of primary AT/RT, such as mesenchymal, epithelial, or rhabdoid architectures [34] being less prominent (Fig. 2c, g). The number of mitotic figures was significantly increased in recurrences (*p* = 0.04, Fig. 2a, c, e, g, i). Ki67 staining resulted in a significantly intensified labeling in recurrences, with an average of approximately 48% Ki67-positive nuclei, compared to 34% in primary tumors (*p* = 0.017, Fig. 2b, d, f, h, j). This suggests that recurrent tumors undergo changes that may be related to additional molecular alterations and even more aggressive tumor growth.

### Epigenetic profiles and molecular type and subtype affiliations remain stable in most AT/RT recurrences

**Fig. 3:**
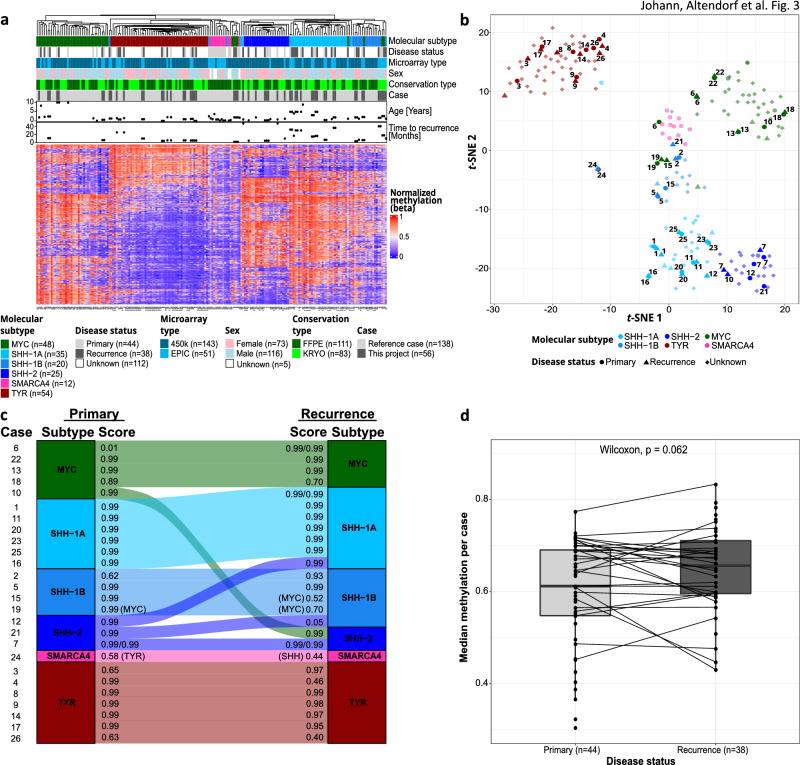
DNA methylation patterns of primary and recurrent AT/RT. **a** Unsupervised hierarchical clustering of matched AT/RT primary tumors and recurrences and reference cases (*n* = 138) based on the 1000 most variable CpG sites. Case numbers indicate patients as listed in Table 1 and Supplementary Table 1. Mostly, primary tumors and recurrences clustered in the same molecular type or subtype independent of time to recurrence. **b** *t*-SNE plot of methylation signature using the 1000 most variable CpG sites. Position of samples mostly suggests similarity between primary and recurrent tumor data. Furthermore, samples predominantly clustered in accordance to the brain tumor classifier disease subgroup results (v12.5, www.molecularneuropathology.org). Transparent dots are the reference cases and not annotated. **c** Alluvial plot of molecular type and subtype alterations in second neoplasms compared to primary tumors according to heatmap and *t*-SNE (**a**, **b**). The cases 10, 12, and 21 changed their molecular type or subtype. Classifier scores were calculated using brain tumor classifier (v12.5, www.molecularneuropathology.org) for AT/RT-SHH, AT/RT-MYC and AT/RT-TYR. According to classifier results, case 19 and the recurrence of case 15 belonged to AT/RT-MYC, but unsupervised hierarchical clustering and *t*-SNE (**a**, **b**), assigned them to AT/RT-SHH-1B. In the following analyses they are annotated as AT/RT-SHH-1B. AT/RT-SHH subtypes AT/RT-SHH-1A, AT/RT-SHH-1B, and AT/RT-SHH-2 were assigned based on *t*-SNE analysis. Case 24 results in low scores for other molecular types, since AT/RT-SMARCA4 is unknown by the brain tumor classifier. Cases 1, 6, and 7 included two recurrences and case 7 also two primary tumors. **d** Median methylation per case based on all measured CpG sites was calculated. Globally, primary and recurrent tumor levels did not differ significantly (Wilcoxon-test; *p* = 0.062)

The normalized DNA methylation values for the 1000 most variable CpG sites was visualized for each primary and second neoplasm of the 26 patients. To show how these cases fit into the landscape of established AT/RT subtypes, we also included a large cohort of reference cases from all known molecular types (*n* = 138, Fig. 3a, b). Unsupervised hierarchical clustering and *t*-SNE analysis of these data demonstrated six clusters matching the molecular types AT/RT-SHH, AT/RT-TYR, AT/RT-MYC, and AT/RT-SMARCA4 and subtypes AT/RT-SHH-1A, AT/RT-SHH-1B, AT/RT-SHH-2. Primary tumors and recurrences were distributed homogeneously over these types and subtypes and did not form their own clusters, indicating that epigenetic changes were subtle and that the profile of a recurrent tumor remained similar to its primary match in most cases. All but three cases showed close neighborhood of primary and recurrent tumors in the unsupervised hierarchical clustering (Fig. 3a) and *t*-SNE analysis (Fig. 3b), which also shows that most AT/RT did not change their molecular type or subtype based on DNA methylation profiling. However, the primary tumor of case 10 belonged to ATRT–MYC and the lesion after second surgery to ATRT–SHH-2, which was also confirmed by brain tumor classifier (v12.5, www.molecularneuropathology.org, [7]). The second neoplasms of cases 12 and 21 belonged to a different subtype within the ATRT–SHH type (Fig. 3c). All three patients suffered from RTPS1.

On DNA methylation level, no significant correlation between primary-recurrence variation and time to recurrence was observed (Supplementary Fig. 1). The primary-recurrence variation was defined as the Jensen–Shannon divergence (JSD) between recurrences and related primary tumors based on the 1000 CpG sites with the largest standard deviation.

We further did not detect significant changes in the global median DNA methylation of primary tumors and related recurrences after integration of our cohort into a data set with unmatched primary (*n* = 17) and recurrent (*n* = 9) AT/RT, regardless of the molecular type (Supplementary Fig. 2). This observation remained stable when evaluating all AT/RT samples together (*p* = 0.062, Fig. 3d).

In conclusion, the majority of the AT/RT recurrences was grossly similar compared to their related primary tumors on DNA methylation level. Three out of 26 AT/RT had a different molecular type or subtype after second surgery, whereas 23 recurrences retained their type/subtype and clustered with their matched primary tumors.

### CNV analysis reveals enhanced chromosome 1q gain and loss of chromosome 10 in AT/RT recurrences

**Fig. 4:**
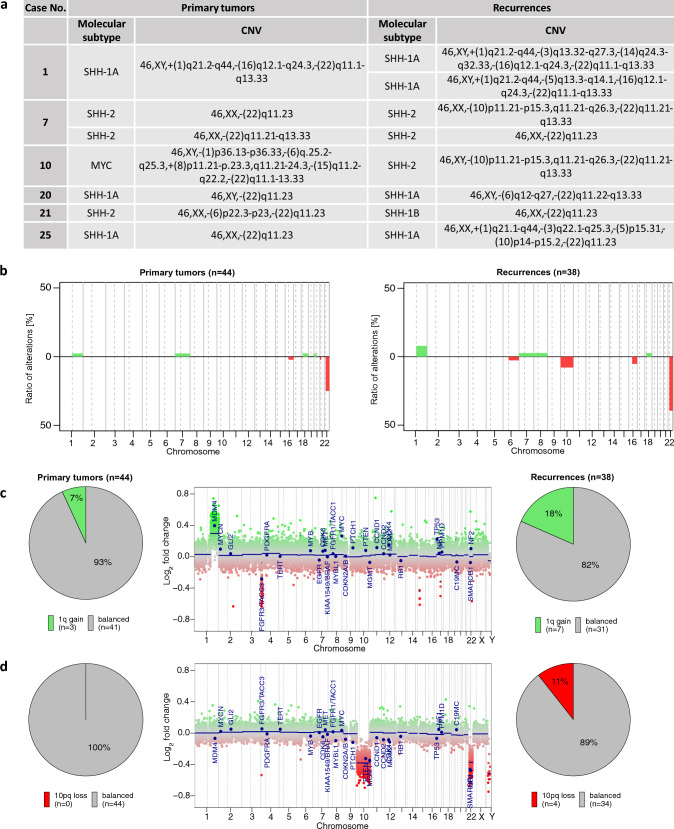
Overview of all cases with different methylation-derived copy number variation (CNV) of primary (*n* = 44) and recurrent (*n* = 38) AT/RT. Individual copy number profiles were calculated based on raw methylated/unmethylated signals using *conumee* package in “R Studio”*.* Profile segments were defined by the span of the chromosome arms. To define, which ratio of methylated-to-unmethylated signal is considered as copy number gain or loss, ratios were normalized against reference samples and cutoff using the local (arm segment) threshold 0.25 and − 0.25, respectively. **a** Overview of cases of our cohort with different CNV of matched primary and recurrent AT/RT. **b** Cumulative CNV of primary tumors (*n* = 44) and recurrences (*n* = 38). **c** Fraction of primary and recurrent tumor samples that showed chromosome 1q gain. Chromosome 1q gain was more often observed in recurrences (18%, *n* = 7) compared to primary tumors (7%, *n* = 3). Exemplary CNV plot showed chromosome 1q gain in the primary tumor of case 15. **d** Fraction of primary and recurrent tumor samples that showed chromosome 10 loss. Chromosome 10 loss was only observed in recurrences (11%, *n* = 4), but not in primary tumors (0%, *n* = 0). Exemplary CNV plot showed chromosome 10 loss in the first recurrence of case 7

CNV analysis was performed to identify further differences between AT/RT primaries and recurrences. CNVs of the recurrences were in general similar to matched primary tumors. Nevertheless, there was a number of cases with different CNVs. The recurrences of cases 1, 7, 10, 20, 21, and 25 showed altered CNVs (Fig. 4a), which included two of the cases that changed their molecular type or rather subtype (cases 10 and 21; Fig. 3b, c). This highlights molecular differences between primary and recurrent tumors for these cases. One of the most common alterations in our cohort was chromosome 1q gain (Fig. 4b), which was confirmed in 7% of primary tumors (n = 3/44) and in 18% of recurrences (n = 7/38; Fig. 4c) after integration of our cohort into the above-mentioned data set of unmatched primary and recurrent AT/RT. Thus, a novel chromosome 1q gain was observed in 11% of the recurrences (Fig. 4b, c), and a loss of chromosome 10 was observed in four out of 38 cases (11%) of all recurrences, but not in primary tumors (Fig. 4b, d). Nearly all copy number variations of our cohort were identified in the AT/RT-SHH types, whereas the other molecular groups seemed to have stable CNVs (Fig. 4a). In general, a loss of chromosome 22q was observed in most cases of all molecular types, which is associated with the *SMARCB1* loss. Other cases had a focal deletion on chromosome 22q, also affecting *SMARCB1*.

Altogether, differences were observed in the CN profile of chromosomes 1q and 10 for AT/RT-SHH recurrences (Supplementary Fig. 3). Genes located in chromosomes 1q and 10 should be further investigated as potential prognostic biomarkers for AT/RT-SHH recurrences.

### Primary tumors and matched recurrences maintain stable immune cell infiltrates but demonstrate decreased infiltration with myeloid dendritic cells in AT/RT-SHH

As is well-known, the environment of a tumor supports its growth and metastatic spread [13, 20]. We analyzed the immune cell infiltration of primary and recurrent AT/RT by calculating the DIMEimmune scores from DNA methylation data [23] and scores from RNA sequencing data using a deconvolution method optimized for brain tumors [4, 5]. These scores were first compared in all AT/RT (Supplementary Fig. 4). This revealed stable immune cell infiltration. When analyzing each of the molecular types separately, infiltration with myeloid dendritic cells was significantly downregulated in AT/RT-SHH tumors (*p* = 0.048, Supplementary Fig. 5j). For AT/RT-TYR, the sample size (*n* = 2) was too small to achieve meaningful results (Supplementary Fig. 6), whereas the immune cell infiltration for AT/RT-MYC did not differ significantly in primaries and recurrences (Supplementary Fig. 7).

### RNA sequencing analysis reveals differentially expressed genes in primary and recurrent AT/RT

**Fig. 5:**
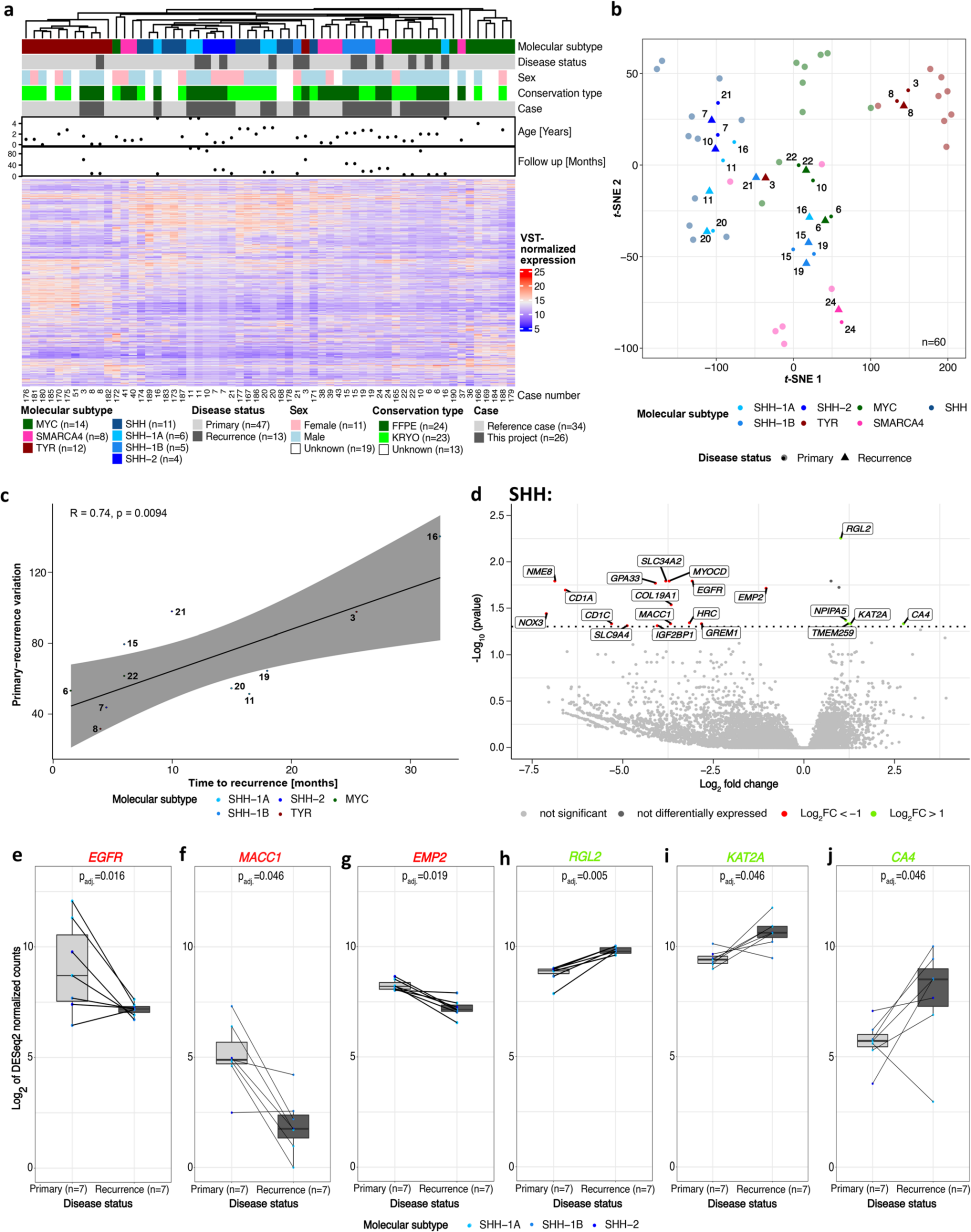
RNA sequencing analysis of primary and recurrent AT/RT. Case numbers indicate patients as listed in Table 1 and Supplementary Tables 1 and 2. Raw RNA sequencing data were processed using *DESeq2* and normalized using variance stabilized transformation (VST) in R Studio. Batch effects introduced by conservation type and microarray type were corrected using the ComBat_Seq method. **a** Unsupervised hierarchical clustering of matched AT/RT primary tumors and recurrences and reference cases (*n* = 33) based on the 1000 most variable genes. Mostly, primary tumors and recurrences clustered in the same molecular type or subtype. The assignment of the molecular types and subtypes was taken from DNA methylation results. **b** *t*-SNE plot using the 1000 most variable genes. Position of samples mostly suggests similarity between primary and recurrent tumor data. Furthermore, samples predominantly clustered in accordance to the brain tumor classifier disease subgroup results. The transparent dots are the reference cases and are not annotated. **c** Positive correlation (*R* = 0.74, *p* = 0.0094) of primary-recurrence variation and time to recurrence [months]. Primary-recurrence variation was defined as the Jensen–Shannon divergence (JSD) between recurrences and related primary tumors based on the 1000 most variable genes. **d** Volcano plot of differentially expressed genes between primary and recurrent AT/RT. Significant genes with a log_2_ fold change > 1 are colored in red for upregulated genes in primary tumors and in green for upregulated genes in recurrences. Highlighted genes were selected for further analysis. **e**–**j** *DESeq2*-normalized counts of paired primary and recurrent AT/RT for *EGFR* (**e**), *MACC1* (**f**), *EMP2* (**g**), *RGL2* (**h**), *KAT2A* (**i**), and *CA4* (**j**). Statistical analysis was performed using *DESeq2*

Normalized expression of the 1000 most variable genes was visualized for each case in a heatmap (Fig. 5a) and a *t*-SNE plot (Fig. 5b) together with reference data (*n* = 33). As for DNA methylation, both analyses resulted in clusters matching more or less the molecular types and subtypes with equal distributions of primary tumors and recurrences. The switch of the molecular type or rather subtype for cases 10 and 21, identified by DNA methylation analysis earlier, was observed in RNA expression-based clustering again. In addition, the recurrence of case 3 was not located in the AT/RT-TYR cluster anymore but switched to AT/RT-SHH. The recurrence of case 16 was located in the AT/RT-SHH-1B cluster and not in the AT/RT-SHH-1A cluster in contrast to DNA methylation data (Fig. 3a, b). Variation of primary tumors and recurrences was positively correlated with the time to recurrence (*R* = 0.74, *p* = 0.0094, Fig. 5c). Recurrences which did not cluster together with their related primary tumors in the heatmap and *t*-SNE plot, had a high primary-recurrence variation and these tumors had a long time to recurrence compared to tumors with smaller variations.

Analysis of differentially expressed genes between primary and recurrent AT/RT was performed for each molecular type, separately, using paired data of 13 patients. This resulted in distinct genes and gene sets. Most of the 13 analyzed patients belonged to the AT/RT-SHH type (*n* = 7, Fig. 5d). After correction for multiple testing, upregulated genes in recurrences were *RGL2*, *NPIPA5*, *TMEM259*, *KAT2A*, and *CA4*. In primary tumors, *EGFR*, *MACC1,* and *EMP2* were upregulated, next to 13 other genes. Three up- or downregulated genes were chosen for further comparison of the log_2_ of *DESeq2*-normalized count data between primary and recurrent AT/RT (Fig. 5e–j), confirming that there were significant differences. In summary, only 21 genes were differentially expressed when comparing primary and recurrent AT/RT-SHH tumors, making a pathway analysis less meaningful. A large number of differentially expressed genes was identified in AT/RT-MYC tumors (Supplementary Fig. 8a), which were further investigated using pathway analyses. This revealed cell cycle-associated genes, HDACs deacetylate/HATs acetylate histones, and genes involved in general metabolism. Immune system-associated pathways and genes were downregulated in recurrences compared to primary tumors (Supplementary Fig. 8b). Analyses of AT/RT-TYR cases resulted in an array of differentially expressed genes, but results differed from AT/RT-MYC (Supplementary Fig. 8c). On one hand, genes involved in signaling by receptor tyrosine kinases and transmission across chemical synapses as well as genes associated with the neuronal system showed increased expression in recurrences. On the other hand, genes involved in developmental processes, such as the development of the neuronal system, including ROBO receptor signaling, were downregulated in recurrences. Disease- and stress-related genes and genes involved in the immune system were downregulated (Supplementary Fig. 8d).

## Discussion

Our analyses demonstrate both, differences and commonalities between AT/RT primary tumors and recurrences on a morphological and molecular level. Histology revealed an enhanced mitotic activity in recurrences, which could be associated with enhanced tumor growth.

By analyzing DNA methylation data of 26 primary tumors and matched recurrences, stable global DNA methylation was observed for most cases. In addition, recurrences mostly belonged to the same methylation class as the related primary tumors. For three out of the 26 recurrences, the methylation class was different, suggesting that they show molecular alterations compared to primary tumors.

A limited number of studies investigate the stability of molecular type allocation in recurrent tumors: for medulloblastomas, it has recently been observed that in a cohort of 57 patients, 56 retained their molecular type upon recurrence [21]. Similarly, Zhao et al. detected a relatively stable methylation phenotype in ependymoma recurrences compared to the respective primaries [32]. Thus, the observed frequency might be in the range of what is to be expected.

As an alternative explanation for these mismatches, it is also possible that these lesions were metachronous tumors in the context of a tumor predisposition. Multiple AT/RT of different molecular types have previously been observed in patients with rhabdoid tumor predisposition syndrome, suffering from synchronous/metachronous or secondary tumors [25, 28]. Indeed, patients #10, 12, and 21 suffered from a rhabdoid tumor predisposition syndrome 1 (RTPS1) due to pathogenic *SMARCB1* germline variants that may have caused multiple independent tumor lesions, but the five other cases with RTPS1 did not demonstrate multiple independent tumors. Though, it was already assumed that patient #10 did not suffer from two independent primary tumors, indicating a clonal origin of the tumors [19]. The recurrence of patient #12 was distant from the primary tumor on a molecular level and, together with the underlying pathogenic germline variant, it may be a metachronous tumor. Recurrences with the same molecular type or rather subtype as their matched primary tumors clustered together, which led to the initial assumption that they seem to have similar DNA methylation patterns. Therefore, CNVs based on DNA methylation data were analyzed as well.

CNVs may influence tumor predisposition meaning that different CNVs could lead to different tumor progression [18]. Therefore, the CNVs of AT/RT primaries and recurrences were compared together with a number of unmatched primary and recurrent AT/RT. Remarkable differences were an increased chromosome 1q gain (11%, *n* = 4) and an enhanced chromosome 10 loss (11%, *n* = 4) in recurrences as compared to primaries. So far, chromosome 1q gain has been associated with an inferior prognosis in different studies of ependymomas [1, 12] and nephroblastomas [2]. Its role in AT/RT deserves further investigation. This is particularly important as chromosome 1q gain is known to induce overexpression of the *MDM4* gene, which contains a p53 tumor suppressor binding domain and inhibits its activity. It may thus represent a potential prognostic marker in AT/RT recurrences.

In addition to general CNV pattern changes between primaries and recurrences, CNV analysis further revealed differences between AT/RT molecular types. CNV alterations, including chromosome 1q gain and chromosome 10 loss, were almost only detected in the AT/RT-SHH type. AT/RT-TYR, AT/RT-MYC, and AT/RT-SMARCA4 showed similar CNVs in primary tumors and recurrences. Despite a generally high homogeneity in the CNV inventory of primaries and recurrences, these differences in AT/RT-SHH merit further investigation.

RNA sequencing analysis revealed differentially expressed genes of AT/RT primary tumors and recurrences. Most of the cases belonged to AT/RT-SHH but, nevertheless, only few genes were significantly differentially expressed compared to AT/RT-MYC and AT/RT-TYR. This could be due to the heterogeneity within the AT/RT-SHH type, which has been shown to be further divided into three subtypes. These AT/RT-SHH subtypes could not be investigated independently due to the small sample size. Nevertheless, the results of the AT/RT-SHH type were most meaningful, whereas the patient number for AT/RT-MYC (*n* = 3) and AT/RT-TYR (*n* = 2) cases were very small. There remains an urgent need to analyze more samples of these molecular types to consolidate our findings.

Five genes were upregulated in AT/RT-SHH recurrences: *RGL2*, *NPIPA5*, *TMEM259*, *KAT2A*, and *CA4*. While the biological function of these genes seems to be diverse, some of these (such as *RGL2*) have been ascribed pro-metastatic functions in other tumor entities [6, 27, 33]. Further studies are necessary to corroborate their biological role in AT/RT-SHH recurrences. The same holds true for 16 genes that were overexpressed in AT/RT-SHH primaries.

For AT/RT-MYC and AT/RT-TYR, there was a higher number of differentially expressed genes. Therefore, a pathway analysis was performed to identify pathways and gene sets, to which the differentially expressed genes were assigned. In general, the sample sizes were small for both with *n* = 3 for AT/RT-MYC and *n* = 2 for AT/RT-TYR. While a statistically sound analysis for AT/RT-TYR was precluded by small sample size, AT/RT-MYC results showed an overexpression of cell cycle-associated genes in recurrences (Supplementary Fig. 8b), which matched our observations of increased mitotic activity in recurrences (Fig. 2). Furthermore, HDACs deacetylate and HATs acetylate histones were enriched gene sets in AT/RT-MYC recurrences. So far, imbalances in the expression of HATs and HDACs have been associated with tumor development and they are known therapeutic targets [22]. Signalling by receptor tyrosine kinases and transmission across chemical synapses were upregulated pathways in AT/RT-TYR recurrences. To name a particularly salient example of an overexpressed gene, *GABRG2*, which encodes a Gamma-Aminobutyric Acid (GABA) receptor, was overexpressed with a log_2_ fold change of about 20, which was much higher compared to all other genes, making it an interesting target for further study.

In conclusion, primary and recurrent AT/RT differed in their histology. An increased mitotic activity and a loss of architecture were observed in recurrences. However, global DNA methylation pattern were very similar for primary and recurrent AT/RT. Most recurrences clustered close to their matching primary tumors and only three out of 26 cases belonged to a different molecular type or subtype after second surgery. Nevertheless, CNV analysis revealed an increased chromosome 1q gain and chromosome 10 loss, mainly in AT/RT-SHH recurrences. Furthermore, 21 differentially expressed cancer-associated genes were identified in AT/RT-SHH recurrences. These genes could be interesting candidates for specific therapeutic targets for this type and should be further investigated in vitro and in vivo. Enriched pathways and gene sets were also identified in AT/RT-MYC and AT/RT-TYR recurrences but need to be further consolidated by more samples.

## Supplementary Information

Below is the link to the electronic supplementary material.

Supplementary file1 (XLSX 31 KB)

Supplementary file2 (PDF 7497 KB)
