# Supplementary material for: Recurrent atypical teratoid/rhabdoid tumors (AT/RT) reveal discrete features of progression on histology, epigenetics, copy number profiling, and transcriptomics
Source: Acta Neuropathol. 2023 Jul 14;146(3):527–41. doi: 10.1007/s00401-023-02608-7 (PMC10412492; doi:10.1007/s00401-023-02608-7)
Supplement: Supplementary file 2 — Supplementary file2 (PDF 7497 KB) [file 401_2023_2608_MOESM2_ESM.pdf]

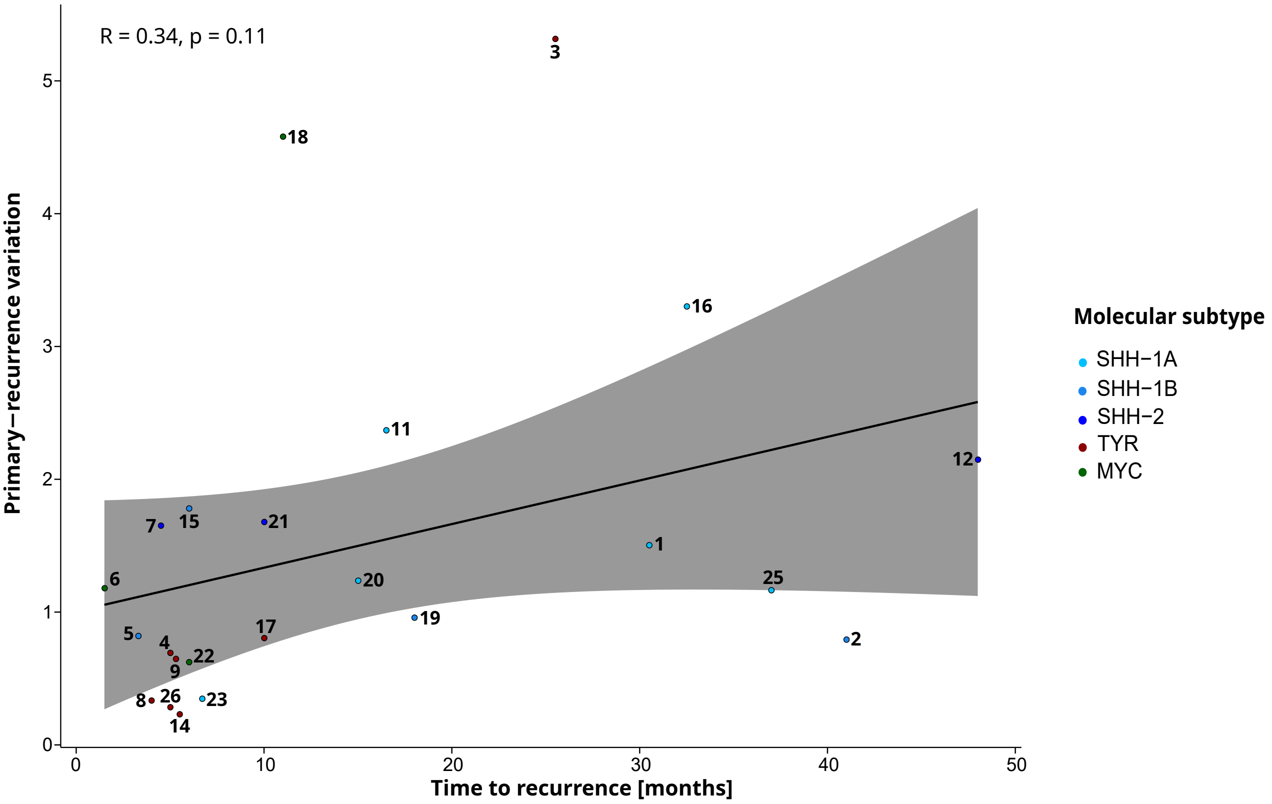

Supplementary Fig. 1: Correlation of primary-recurrence variation and time to recurrence based on DNA methylation data. Primary-recurrence variation was defined as the Jensen-Shannon divergence between primary tumors and related recurrences based on the 1,000 CpG sites with the largest standard deviation. No correlation was observed on DNA methylation level.

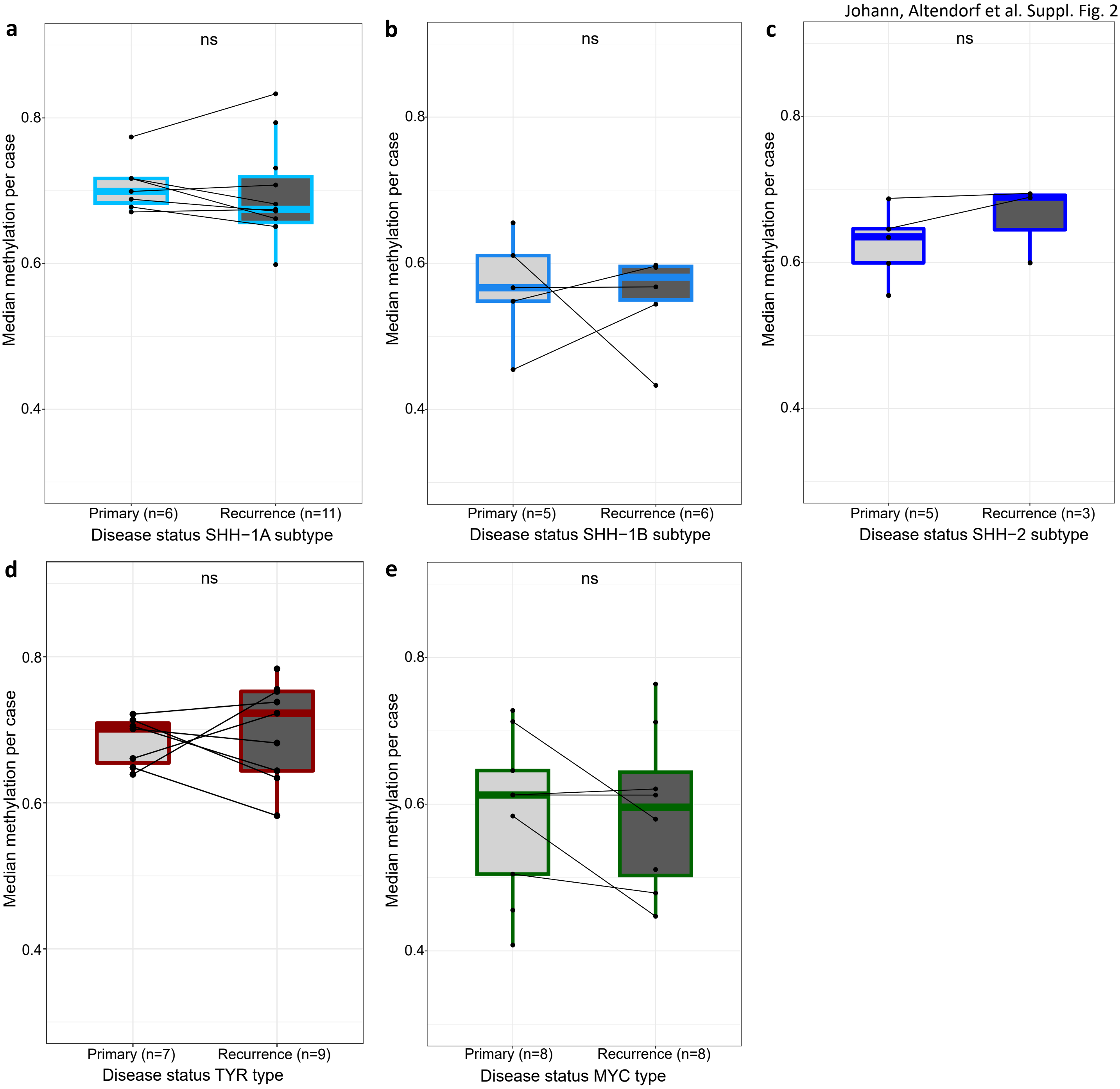

Supplementary Fig. 2: Global DNA methylation patterns of primary and recurrent AT/RT for each molecular type and subtype. Median methylation per case for each molecular type and subtype based on all CpG sites was calculated. **a - e** No significant differences of median methylation per case between disease states can be detected for AT/RT-SHH-1A (**a**), AT/RT-SHH-1B (**b**), AT/RT-SHH-2 (**c**), AT/RT-TYR (**d**), and AT/RT-MYC (**e**). Significance of methylation levels between primary and recurrent tumors was determined using a Wilcoxon test.

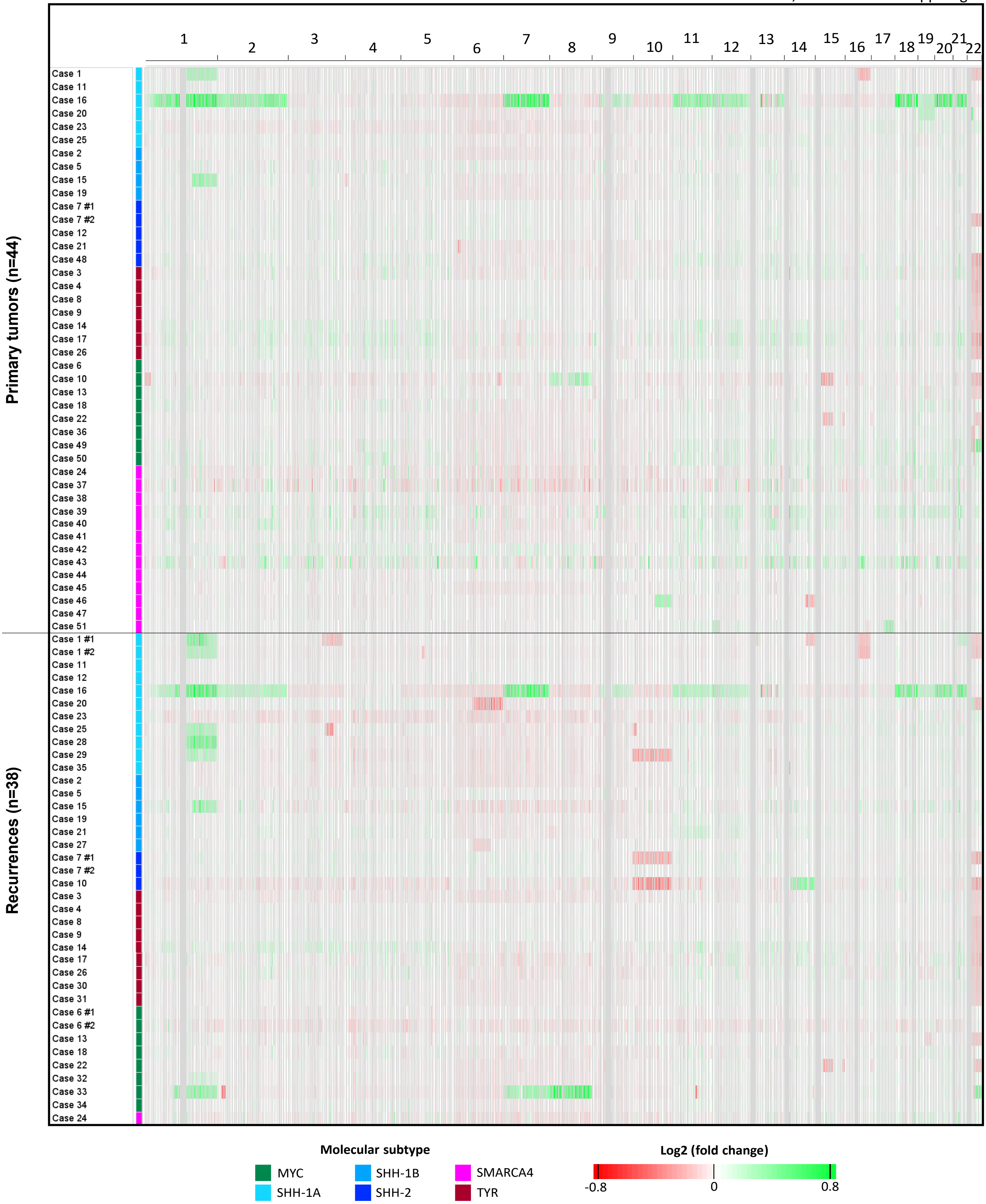

Supplementary Fig. 3: Methylation-derived copy number variation (CNV) of all primary (n=44) and recurrent (n=38) AT/RT. Individual copy number (CN) profiles were created using Integrative Genomics Viewer "IGV" based on raw methylated/ unmethylated signals. Green indicates a CN gain and red a CN loss. CNV profiles were sorted by disease status.

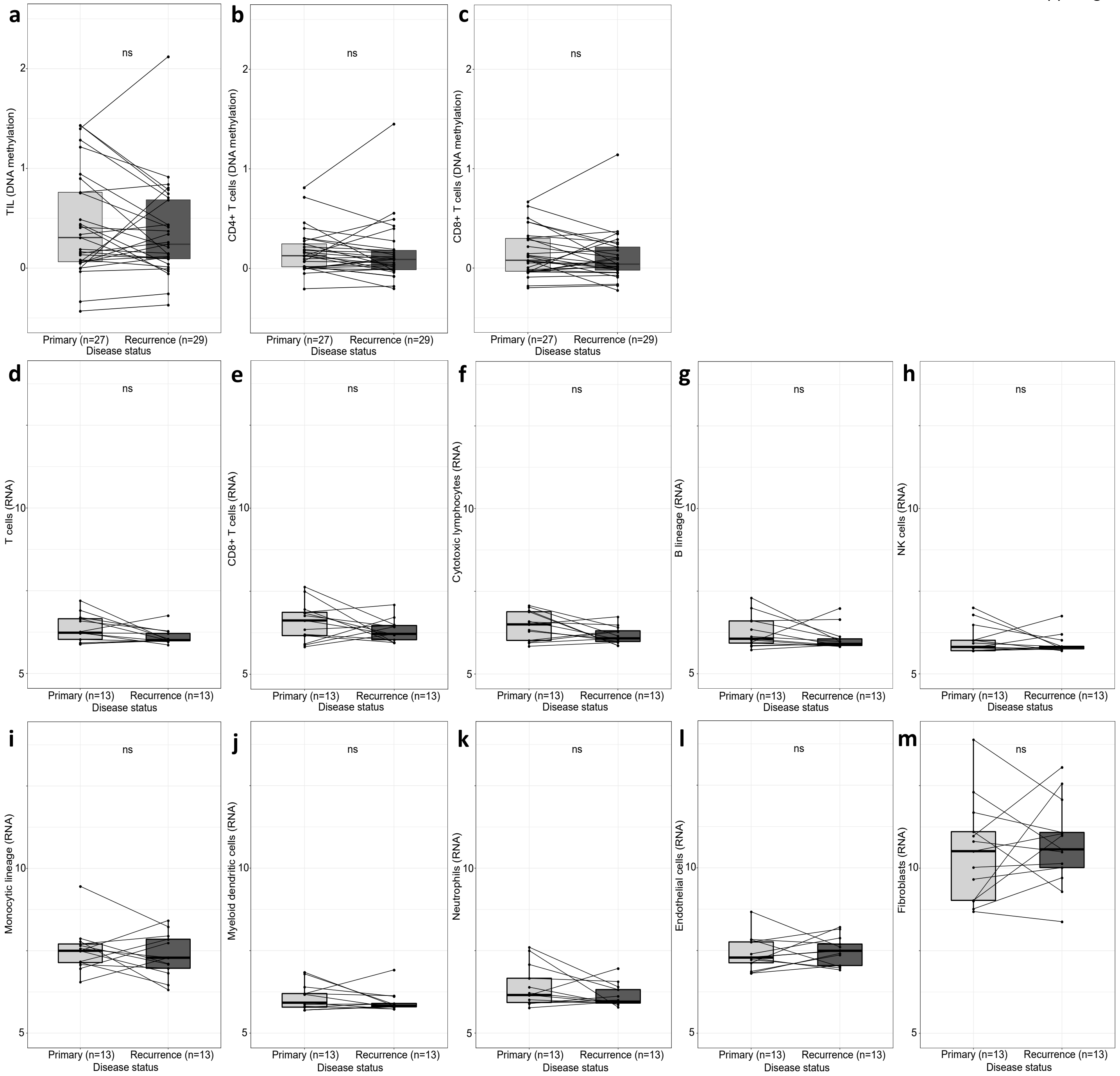

Supplementary Fig. 4: Estimation of immune cell infiltrates in primary tumors compared to recurrences of AT/RT. a - c DIMEimmune scores for infiltration with tumor-infiltrating lymphocytes (TILs) (**a**), CD4$^+$ T cells (**b**), and CD8$^+$ T cells (**c**) were normalized and calculated according to Safaei et al. (2021) using DNA methylation data. Each primary tumor was paired with related recurrence. Statistical analysis was performed using a t-test for paired data. **d - m** Scores for ten additional cell populations, T cells (**d**), CD8$^+$ T cells (**e**), cytotoxic lymphocytes (**f**), B lineage cells (**g**), NK cells (**h**), monocytic lineage cells (**i**), myeloid dendritic cells (**j**), neutrophils (**k**), endothelial cells (**l**), and fibroblasts (**m**), were calculated using RNA sequencing data according to Bockmayr et al. (2018, 2019). Each primary tumor was paired with related recurrence. Statistical analysis was performed using a t-test for paired data.

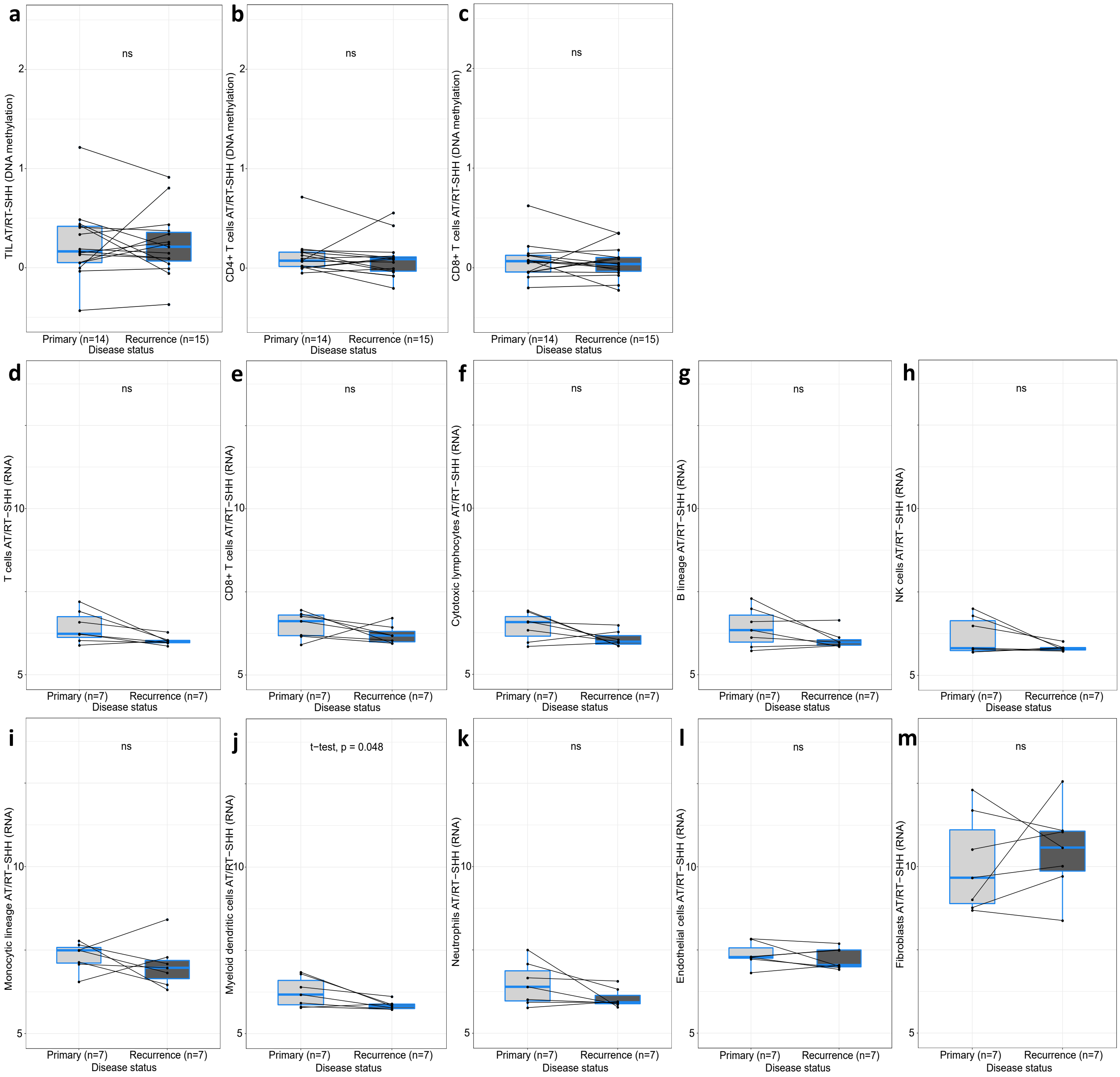

Supplementary Fig. 5: Estimation of immune cell infiltrates in primary tumors compared to recurrences of AT/RT-SHH. a - c DIMEimmune scores for infiltration with tumor-infiltrating lymphocytes (TILs) (**a**), $CD4^+$ T cells (**b**), and $CD8^+$ T cells (**c**) were normalized and calculated according to Safaei et al. (2021) using DNA methylation data. Each primary tumor was paired with related recurrence of the SHH subtype. Statistical analysis was performed using a t-test for paired data. **d - m** Scores for ten additional cell populations, T cells (**d**), $CD8^+$ T cells (**e**), cytotoxic lymphocytes (**f**), B lineage cells (**g**), NK cells (**h**), monocytic lineage cells (**i**), myeloid dendritic cells (**j**), neutrophils (**k**), endothelial cells (**l**), and fibroblasts (**m**), were calculated using RNA sequencing data according to Bockmayr et al. (2018, 2019). Each primary tumor was paired with related recurrence of the SHH subtype. Statistical analysis was performed using a t-test for paired data.

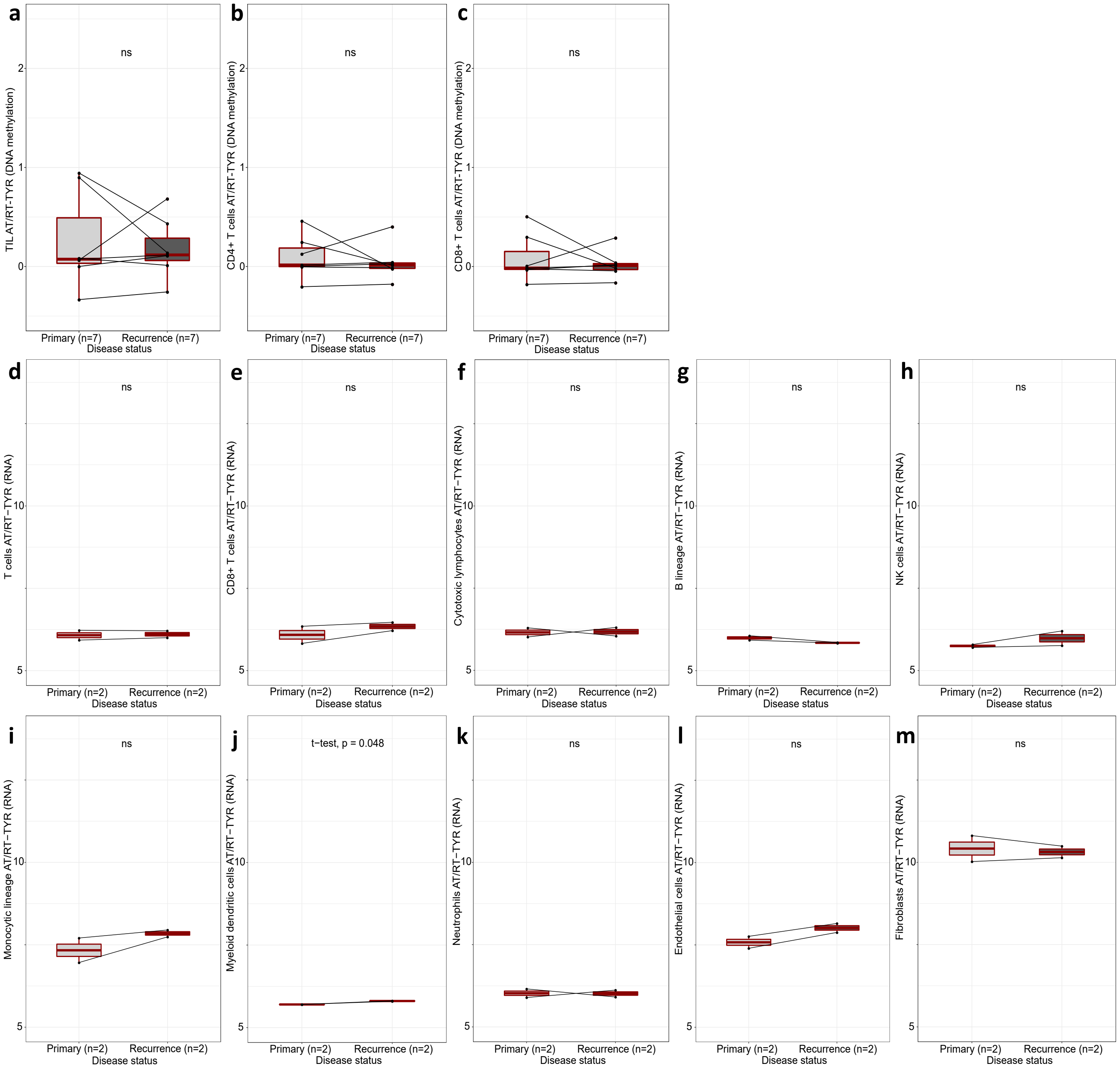

Supplementary Fig. 6: Estimation of immune cell infiltrates in primary tumors compared to recurrences of AT/RT-TYR. a - c DIMEimmune scores for infiltration with tumor-infiltrating lymphocytes (TILs) (**a**), CD4$^+$ T cells (**b**), and CD8$^+$ T cells (**c**) were normalized and calculated according to Safaei et al. (2021) using DNA methylation data. Each primary tumor was paired with related recurrence of the TYR subtype. Statistical analysis was performed using a t-test for paired data. **d - m** Scores for ten additional cell populations, T cells (**d**), CD8$^+$ T cells (**e**), cytotoxic lymphocytes (**f**), B lineage cells (**g**), NK cells (**h**), monocytic lineage cells (**i**), myeloid dendritic cells (**j**), neutrophils (**k**), endothelial cells (**l**), and fibroblasts (**m**), were calculated using RNA sequencing data according to Bockmayr et al. (2018, 2019). Each primary tumor was paired with related recurrence of the TYR subtype. Statistical analysis was performed using a t-test for paired data.

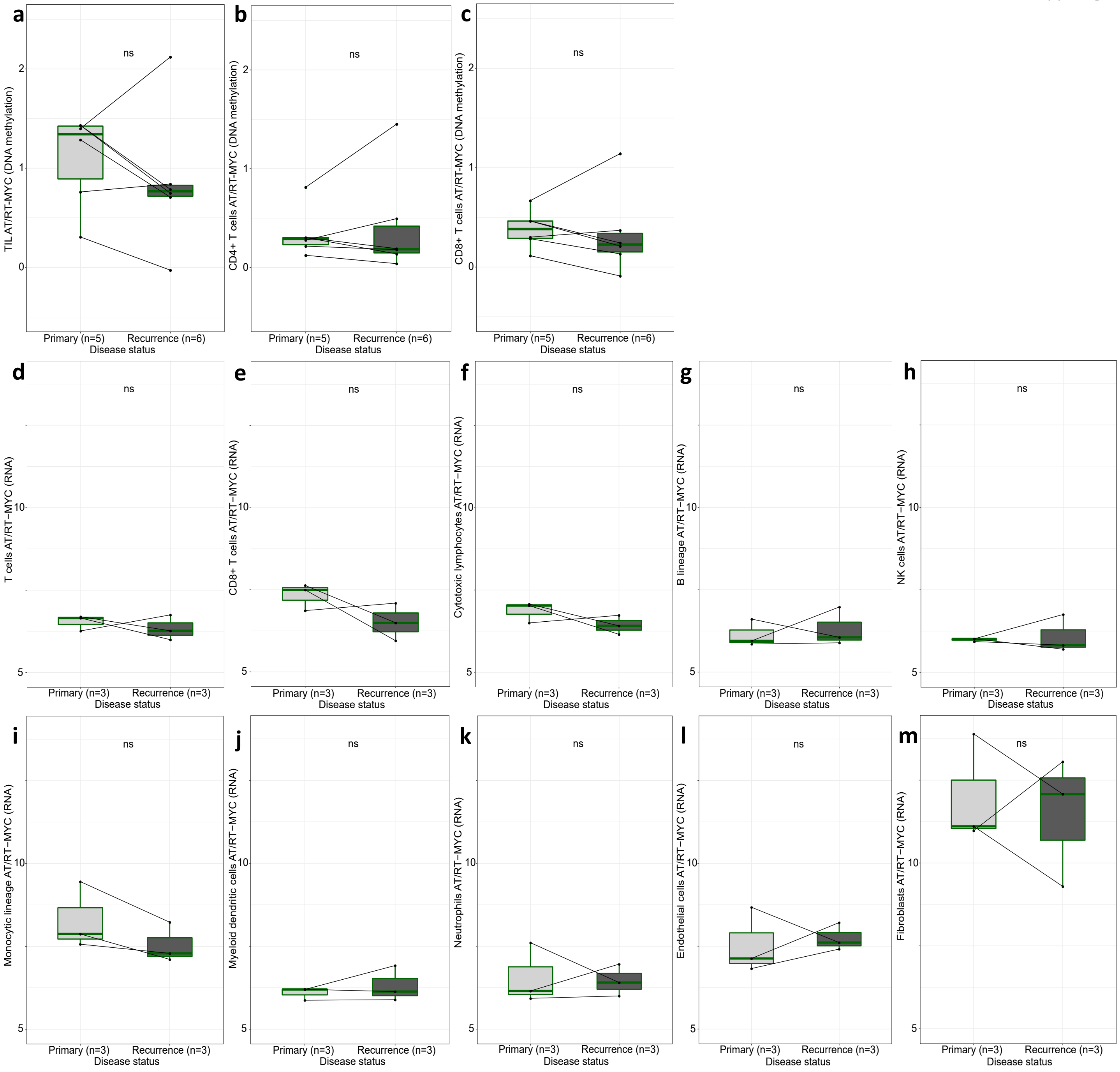

Supplementary Fig. 7: Estimation of immune cell infiltrates in primary tumors compared to recurrences of AT/RT-MYC. a - c DIMEimmune scores for infiltration with tumor-infiltrating lymphocytes (TILs) (**a**), CD4^{+} T cells (**b**), and CD8^{+} T cells (**c**) were normalized and calculated according to Safaei et al. (2021) using DNA methylation data. Each primary tumor was paired with related recurrence of the MYC subtype. Statistical analysis was performed using a t-test for paired data. **d - m** Scores for ten additional cell populations, T cells (**d**), CD8^{+} T cells (**e**), cytotoxic lymphocytes (**f**), B lineage cells (**g**), NK cells (**h**), monocytic lineage cells (**i**), myeloid dendritic cells (**j**), neutrophils (**k**), endothelial cells (**l**), and fibroblasts (**m**), were calculated using RNA sequencing data according to Bockmayr et al. (2018, 2019). Each primary tumor was paired with related recurrence of the MYC subtype. Statistical analysis was performed using a t-test for paired data.

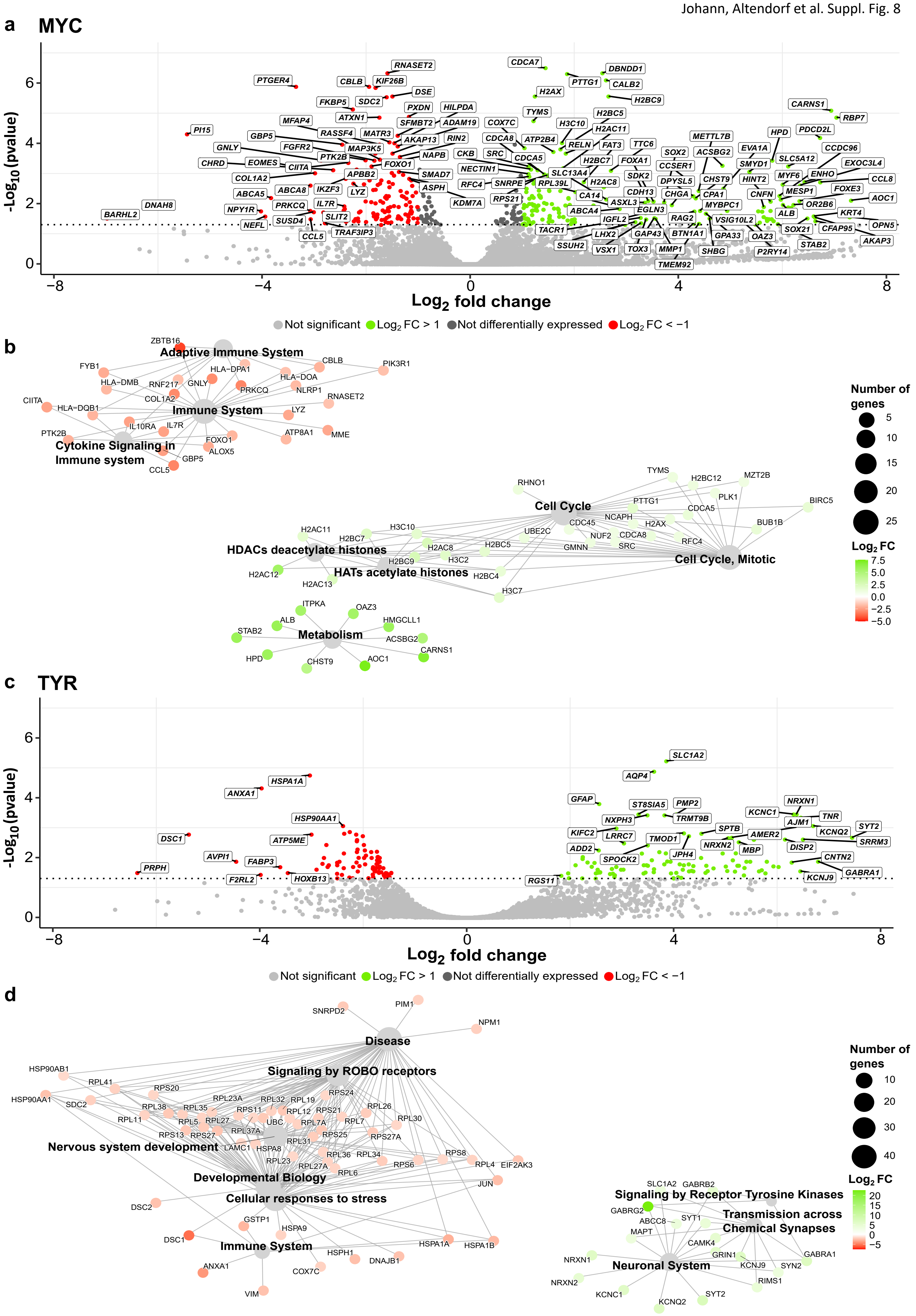

a MYC
-Log10 (pvalue)
Log2 fold change
Not significant
Log2 FC > 1
Not differentially expressed
Log2 FC < −1
RNASET2
PTGER4
CBLB
KIF26B
DSE
FKBP5
SDC2
PXDN
HILPDA
MFAP4
ATXN1
SFMBT2
ADAM19
PI15
GBP5
RASSF4
MATR3
AKAP13
RIN2
GNLY
FGFR2
MAP3K5
NAPB
CKB
CHRD
EOMES
PTK2B
CIITA
FOXO1
COL1A2
APBB2
SMAD7
ABCA5
ABCA8
IKZF3
LYZ
ASPH
KDM7A
DNAH8
PRKCQ
IL7R
NPY1R
BARHL2
SLIT2
SUSD4
NEFL
CCL5
TRAF3IP3
CDCA7
DBNDD1
PTTG1
CALB2
H2AX
H2BC9
TYMS
H2BC5
COX7C
H3C10
H2AC11
CDCA8
ATP2B4
RELN
FAT3
TTC6
SRC
CDCA5
H2BC7
FOXA1
NECTIN1
SLC13A4
SDK2
RFC4
SNRPE
RPL39L
H2AC8
CDH13
RPS21
CA14
ASXL3
ABCA4
EGLN3
IGFL2
TACR1
LHX2
GAP43
SSUH2
VSX1
TOX3
MMP1
TMEM92
METTL7B
SOX2
ACSBG2
CCSER1
DPYSL5
CHST9
CHGA
CPA1
MYBPC1
RAG2
VSIG10L2
BTN1A1
GPA33
SHBG
CARNS1
RBP7
HPD
PDCD2L
EVA1A
SMYD1
SLC5A12
CCDC96
EXOC3L4
HINT2
MYF6
ENHO
CCL8
CNFN
MESP1
FOXE3
AOC1
ALB
OR2B6
KRT4
OPN5
OAZ3
SOX21
CFAP95
STAB2
AKAP3
P2RY14
b
Adaptive Immune System
Immune System
Cytokine Signaling in Immune system
ZBTB16
FYB1
HLA−DPA1
CBLB
PIK3R1
HLA−DMB
RNF217
GNLY
HLA−DOA
PRKCQ
NLRP1
COL1A2
CIITA
HLA−DQB1
RNASET2
LYZ
IL10RA
IL7R
PTK2B
ATP8A1
MME
FOXO1
ALOX5
GBP5
CCL5
Cell Cycle
Cell Cycle, Mitotic
HDACs deacetylate histones
HATs acetylate histones
RHNO1
TYMS
H2BC12
MZT2B
PLK1
PTTG1
BIRC5
CDCA5
NCAPH
CDC45
H2AX
BUB1B
UBE2C
NUF2
CDCA8
RFC4
GMNN
SRC
H2AC11
H3C10
H2BC7
H2AC8
H2BC5
H2BC9
H3C2
H2AC12
H2AC13
H2BC4
H3C7
Metabolism
ITPKA
OAZ3
ALB
HMGCLL1
STAB2
ACSBG2
CARNS1
HPD
CHST9
AOC1
Number of genes
5
10
15
20
25
Log2 FC
7.5
5.0
2.5
0.0
−2.5
−5.0
c TYR
-Log10 (pvalue)
Log2 fold change
SLC1A2
AQP4
HSPA1A
ANXA1
GFAP
ST8SIA5
PMP2
NRXN1
KCNC1
NXPH3
TRMT9B
TNR
HSP90AA1
AJM1
SYT2
DSC1
KIFC2
LRRC7
SPTB
AMER2
KCNQ2
ATP5ME
TMOD1
SRRM3
ADD2
NRXN2
MBP
DISP2
AVPI1
JPH4
FABP3
SPOCK2
CNTN2
PRPH
GABRA1
F2RL2
HOXB13
RGS11
KCNJ9
d
Disease
Signaling by ROBO receptors
Nervous system development
Developmental Biology
Cellular responses to stress
Immune System
SNRPD2
PIM1
NPM1
HSP90AB1
RPL41
RPS20
HSP90AA1
SDC2
RPL23A
RPL32
RPL19
RPS24
RPL38
RPL35
RPS11
RPL12
RPS21
RPL26
RPL11
RPL5
RPL27
UBC
RPL7A
RPL7
RPL30
RPS13
RPS27
RPL37A
RPS25
RPS27A
RPL31
LAMC1
HSPA8
RPL36
RPL34
RPS8
RPL23
RPL27A
RPS6
RPL4
EIF2AK3
RPL6
JUN
DSC2
GSTP1
HSPA9
DSC1
HSPA1A
HSPA1B
HSPH1
DNAJB1
ANXA1
COX7C
VIM
Signaling by Receptor Tyrosine Kinases
Transmission across Chemical Synapses
Neuronal System
SLC1A2
GABRB2
GABRG2
ABCC8
SYT1
MAPT
CAMK4
GRIN1
GABRA1
NRXN1
KCNJ9
SYN2
NRXN2
RIMS1
KCNC1
KCNQ2
SYT2
Number of genes
10
20
30
40
Log2 FC
20
15
10
5
0
−5

Supplementary Fig. 8: Differentially expressed genes and pathways in AT/RT-MYC and AT/RT-TYR primary tumors and recurrences. a + c Volcano plots of differentially expressed genes between primary and recurrent AT/RT-MYC (**a**) and AT/RT-TYR (**c**). Significant genes with a $\log_2$ fold change > 1 are colored in red for upregulated genes in primary tumors and in green for upregulated genes in recurrences. **b + d** Enriched pathways and genes in AT/RT-MYC (**b**) and AT/RT-TYR (**d**) recurrences. Colors indicate the $\log_2$ FC of the gene expression. Green dots mark a positive $\log_2$ FC and red dots a negative. The size of the pathway dots indicates the number of involved genes in the pathways.
